# The Public Health Services

**Published:** 1918-09-07

**Authors:** 


### THE PUBLIC HEALTH SERVICES.

THE passing of the Great War into the fifth year of its duration serves to emphasise the necessity of directing increased attention to the problem of public health in relation to national welfare and future progress. National development and industrial progress depend on a high individual standard of mental and physical capacity, and this standard can only be attained by the carrying out of adequate and comprehensive measures for the prevention and treatment of disease. This, in great measure, is the duty which devolves on the Public Health Services.

Within recent years the field of work in relation to public health has been largely increased in scope and character. While formerly it was chiefly concerned with the prevention and elimination of known conditions which pave the way for disease, it has now come to include far-reaching measures which have as their aim the early diagnosis and appropriate treatment of certain forms of disease and defects. Thus, since the year 1911 the sphere of activity of Public Health Authorities has come to include the following: the diagnosis and treatment of the various forms of tuberculosis, the ascertainment of, and the provision of institutional accommodation for, mental defectives, the diagnosis and treatment of venereal diseases, the provision of Maternity and Child-Welfare Centres including suitable advice and treatment to mothers and children under the age of five, the completing of adequate and efficient services of midwives and the providing of facilities for the diagnosis and specific treatment of certain acute diseases, such as cerebro-spinal meningitis. In addition, increased efforts have been made to minimise the injurious effects to the health and lives of infants and young children from such diseases as ophthalmia neonatorum, measles, and infantile diarrhœa. In the majority of districts also the Public Health Services embrace the school medical staff, so that the work of the Services includes the medical inspection of school children and the treatment of defects and minor ailments at school clinics. These branches of public health which have recently been developed serve to accentuate the increasing need for central and local unification of control and effort. The highest aims of the Public Health Services can never be realised until the problems relating to national health and the prevention and treatment of disease are unified and co-ordinated under one responsible authority.

While it is impossible to forecast with any degree of accuracy to what extent reconstruction after the war may amplify and extend the work of the Public Health Services, yet there are indications that along certain lines definite development will take place. It is anticipated that there will be increased institutional provision for the treatment of disease and for the training of defectives, greater effort towards conserving the lives and health of mothers, infants, and children, and wider facilities for the early and correct diagnosis of disease by the provision of public health laboratories. In addition, the Poor-Law system will be remodelled on an up-to-date basis so as to bring it into line with modern public health requirements. It will thus be seen that the Public Health Services will offer to the medical practitioner an increasing field for attractive and remunerative work. It is true that in certain branches of the Services the salaries given cannot be regarded as adequate in view of the specialised character of the work done, and that the important questions of security of tenure and the provision of pensions have not as yet been established on a satisfactory basis, but there is no doubt that this will be done in the near future. Apart from these important questions, the Services are becoming increasingly attractive to medical practitioners in view of the interesting and varied character of much of the new work which is now being undertaken.

There are open to the medical practitioner who contemplates undertaking public health work, in the wide modern sense of that term, three separate fields of specialised activity. If his inclination tend towards administration and the organising of schemes, he will find ample scope in the administrative part of public health work; if he prefer clinical work, such is provided in the official schemes for the diagnosis and treatment of disease and defects now in existence. Lastly, if the trend of his mind be towards scientific work and investigation, facilities for such will be found in public health laboratories, provision of which will undoubtedly be made on a much more extended scale after the war. The experience and qualifications necessary to obtain an appointment in the Public Health Services are well known and need only be briefly referred to. After qualification, the holding of a house appointment in a large general hospital and some experience of general practice are to be recommended, as a sound clinical knowledge of disease and the ability to view the problems relating to prevention and treatment from the standpoint of the general practitioner will be found to be of distinct value. A course of special study in public health work and the holding of a diploma in public health or sanitary science are necessary before the higher appointments can be obtained. Ability and industry are, of course, primary qualifications which tell in the Public Health Services as in other walks of life, but in much of the administrative work connected with public health tact and discrimination are not infrequently of greater value than talent.

